# Increased Consumption of Fruit and Vegetables Is Related to a Reduced Risk of Cognitive Impairment and Dementia: Meta-Analysis

**DOI:** 10.3389/fnagi.2017.00018

**Published:** 2017-02-07

**Authors:** Xian Jiang, Jiang Huang, Daqiang Song, Ru Deng, Jicheng Wei, Zhuo Zhang

**Affiliations:** ^1^Department of Anesthesia, The Affiliated Hospital of Southwest Medical UniversityLuzhou, China; ^2^Department of Pharmacy, The Affiliated Traditional Chinese Medicine Hospital of Southwest Medical UniversityLuzhou, China; ^3^Department of Pharmacology, School of Pharmacy of Southwest Medical UniversityLuzhou, China

**Keywords:** fruit, vegetable, cognitive impairment, dementia, meta-analysis

## Abstract

**Background:** Increased consumption of fruit and vegetables has been shown to be associated with a reduced risk of cognitive impairment and dementia in many epidemiological studies. The purpose of this study was to assess the strength of this association in a meta-analysis.

**Methods:** We identified relevant studies by searching Medline, Embase, and Cochrane Library electronic databases (from 1970 to January 2016). Study were included if they reported relative risks and corresponding 95% confidence intervals (CIs) of cognitive impairment and dementia with respect to frequency of fruit and vegetable intake.

**Results:** Nine studies (five cohort studies and four cross-sectional studies) met the inclusion criteria and were included in the meta-analysis. There were a total of 31,104 participants and 4,583 incident cases of cognitive impairment and dementia. The meta-analysis showed that an increased consumption of fruit and vegetables was associated with a significant reduction in the risk of cognitive impairment and dementia (OR = 0.80, 95% CI 0.71–0.89). Subgroup analysis indicated this inverse association was only found among participants with mean age over 65 years and combined sexes. Dose–response meta-analysis showed that an increment of 100 g per day of fruit and vegetable consumption was related to an approximately 13% (OR = 0.87, 95% CI 0.77–0.99) reduction in cognitive impairment and dementia risk. There was no potential publication bias in the meta-analysis and the dose–response meta-analysis.

**Conclusion:** The increased consumption of fruit and vegetables is associated with a reduced risk of cognitive impairment and dementia.

## Introduction

Cognitive impairment is an important public health concern, with a prevalence that is expected to rise with the population aging. It is a transitional stage between normal aging and dementia. Interventions may be particularly effective at earlier stages of disease development, which offer an opportunity for reducing the public health burden of Alzheimer’s disease (AD) and other dementias through early detection and prevention (Morris et al., 1991; Roberts et al., 2010).

A number of epidemiologic researches and animal models have found associations between dietary components and age-related cognitive impairment and dementia (Joseph et al., 2009; Aparicio Vizuete et al., 2010; Gu and Scarmeas, 2011; Bondonno et al., 2014; Wu et al., 2015), and adherence to a Mediterranean-type diet was associated with slower cognitive decline and a reduced risk for AD in elderly individuals (Feart et al., 2009; Scarmeas et al., 2009). Fruit and vegetables are important components of a Mediterranean diet, which are high in antioxidants, vitamins, and folate, and in epidemiological and laboratory studies (Paleologos et al., 1998; Engelhart et al., 2002; Liu et al., 2002), these micronutrients have been related to cognitive benefits. Ye et al. (2013) found high variety of fruit and vegetable consumption was associated with individual cognitive domains, including executive function, memory and attention. A previous review of several cohort studies has examined the association between fruit and vegetable consumption and cognitive decline or dementia (Loef and Walach, 2012). In general, these studies find a favorable relation between fruit and vegetable consumption and risk of dementia or cognitive decline, although sometimes the results are inconsistent. Moreover, the strength of the favorable relation remains uncertain due to the differences in sample selections, methodological approaches, analytical techniques, and outcome definitions. Therefore, we conducted a meta-analysis to quantitatively assess the relation between fruit and vegetable consumption and the risk of cognitive impairment and dementia.

## Materials and Methods

### Search Strategy and Eligibility Criteria

We followed the guidelines published by the Meta-analysis of Observational Studies in Epidemiology (MOOSE) group to complete the meta-analysis (Supplementary Table S1) (Stroup et al., 2000). Two investigators performed a systematic literature search of Medline, Embase, and Cochrane Library electronic databases (from 1970 to January 2016) to identify eligible articles. The electronic search includes both MeSH and free-text terms. The following terms were used: “Fruit,” “Vegetables,” “Mild Cognitive Impairment,” “Dementia,” “cognitive decline,” “cognitive impairment,” “survey,” and “Data Collection.” The references of all retrieved articles and recent reviews were also manually reviewed. The search strategy was not limited by study design. No attempt was made to find articles in languages other than English or to contact authors of unpublished works.

A study was eligible for inclusion if the following criteria were met: (1) examination of dietary consumption of fruit and/or vegetables as the variable of interest; (2) determination of incidence of cognitive impairment or dementia as the outcome of interest; and (3) reporting the relative risks of cognitive impairment or dementia calculated according to the highest category with the lowest category of fruit and/or vegetables consumption, and their 95% confidence intervals (CIs). The studies about animal experiment, mechanistic research, and review research were excluded.

### Data Extraction and Study Quality Evaluation

Two researchers independently extracted the following data from each publication: author, country, study design, sample size, disease type (cognitive impairment or dementia), number of cases, age, disease ascertainment, exposure variable (fruit, vegetable, or fruit and vegetable), exposure assessment, risk estimates with CIs, and factors adjusted for. The most adjusted estimate was included when a study reported more than one risk estimate. The quality of each study was assessed by two researchers, using the Newcastle-Ottawa Scale recommended by Wells et al. (2011).

### Statistical Analysis and Data Synthesis

We performed meta-analyses of risk estimates for cognitive impairment and dementia comparing the highest category of exposure of fruit and vegetables with the lowest category. We pooled the data on cognitive impairment and dementia together, since there were limited studies on each single disease. Dose–response meta-analyses of fruit and vegetable consumption and risk of cognitive impairment and dementia were then conducted using methods previously reported (Greenland and Longnecker, 1992; Larsson and Orsini, 2011), which facilitated the calculation of a pooled relative risk across studies with a common unit of comparison with studies, assuming a linear dose–response relation. In the current study, we estimated the relative risk per unit of 100 g increment of fruit and vegetable consumption per day for each study and then pooled them together. For studies that reported results for fruit and/or vegetable consumption in servings only, we derived grams by assuming that the average serving equals 80 g for fruits and 77 g for vegetables (He et al., 2007). We converted the level of fruit and vegetable consumption categories based on the calculated midpoint of fruit and vegetable consumption if the study did not report the median of exposure category. Supplementary Table S2 shows the definition of fruit and vegetable consumption and the means of conversion of categories within each study.

We used a fixed effects model to estimate the pooled ORs and 95% CI if there was no evidence of heterogeneity; otherwise, a random effect model was used. The *I*-squared (*I*^2^) statistic and *Q*-statistic were used to explore the heterogeneity among studies. Large *I*^2^ (>50%) or *P* < 0.10 for *Q*-statistic suggests substantial heterogeneity among studies. We also performed a sensitivity analysis by removing each individual study from the meta-analysis (Tobias, 1999). Publication bias was visually assessed by using funnel plots. Egger’s regression test (Egger et al., 1997) and Begg–Mazumdar test (Begg and Mazumdar, 1994) were used to further assess publication bias. Subgroup analyses were performed according to the mean age of sample, sex, geographic location, study design, study quality score, disease type, and dietary assessment method. Statistical analyses were conducted using Stata Version 12.0 software (Stata Corp., College Station, TX, USA).

## Results

### Search Results

**FIGURE 1:**
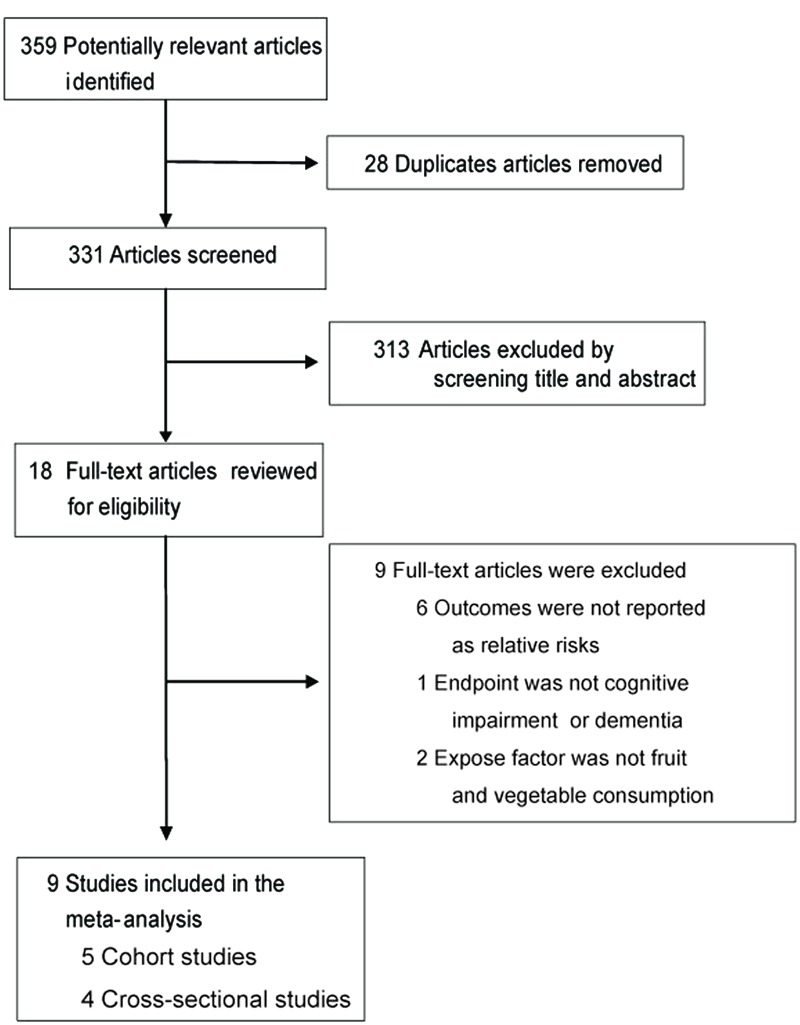
**Flowchart for the selection of eligible studies**.

Of the 359 citations identified from database searches, nine articles met the inclusion criteria and were included in the meta-analysis, including five cohort studies (Barberger-Gateau et al., 2007; Vercambre et al., 2009; Hughes et al., 2010; Ritchie et al., 2010; Chen et al., 2012) and four cross-sectional studies (Lee et al., 2010; Roberts et al., 2010; Wu et al., 2011; Chan et al., 2013). The study selection process is shown in **Figure 1**. Among the included studies, four were from Europe, four were from China, and one was from the United States. **Table 1** summarizes the characteristics of the included studies. The analysis involved a total of 31,104 participants and 4,583 incident cases of cognitive impairment and dementia. Frequency of fruit and vegetable consumption was recorded in different categories from the least frequent consumption to the most frequent consumption. We recorded relative risks of cognitive impairment and dementia according to the highest vs. lowest category of fruit and vegetable consumption. The quality assessment of the included studies was presented in detail in the supplementary material (Supplementary Tables S3 and S4).

**Table 1:** Characteristics of included studies in the systematic review and meta-analysis.

Study	Country	Study design	Sample size	Follow-up duration (years)	Disease type, number of cases	Age (years), mean/range	Disease ascertainment	Exposure variable	Exposure assessment	Factors adjusted for
Barberger-Gateau et al. (2007)	France	Cohort	8,085	3.5	Dementia, 281	NR/≥65	Neurological exam, DSM-IV, and NINCDS-ADRDA	F+V	FFQ	Age, gender, education, city, income, marital status, ApoE genotype, BMI, and diabetes

Chan et al. (2013)	Hong Kong, China	Cross-sectional	3,670	–	Cognitive impairment, 877	72.4/≥65	CSI-D	F+V	FFQ	Age, BMI, PASE, energy intake, educational level, Hong Kong ladder, community ladder, smoking status, alcohol use, No. of ADLs, GDS category, self-reported history of DM, hypertension, and CVD/stroke

Chen et al. (2012)	China	Cohort	5,691	NR	Cognitive impairment, 1,306	82.9/≥65	MMSE	Fruit	Interviewer-administrated questionnaire	None
								Vegetable		Age, gender, marital status, financial status, residential area, BMI, hypertension, diabetes, smoking, alcohol, tea drinking, and exercise habits

Hughes et al. (2010)	Sweden	Cohort	3,779	31.5	Dementia, 335	48.3/42–71	DSM-IV and NINCDS-ADRDA	F+V	The Swedish Twin Registry 1967 questionnaire	Age at cognitive screening, gender, education, smoking, alcohol drinking, angina pectoris, BMI, total food compared to others, marital status, and exercise

Lee et al. (2010)	Hong Kong, China	Cross-sectional	285	–	Dementia, 146	70.5/≥60	DSM-IV and CDR	F+V	Mini-Nutritional Assessment (Chinese version)	Age, sex, and education

Ritchie et al. (2010)	France	Cohort	1,433	7.3	Mild cognitive impairment or dementia, 405	72.5/≥65	Standardized interview incorporating cognitive testing	F+V	Nutritional questionnaires	Age and sex

Roberts et al. (2010)	United States	Cross-sectional	1,233	–	Mild cognitive impairment, 163	NR/70–89	CDR, the Short Test of Mental Status, the Hachinski Scale, and neurological examination.	Fruit and vegetable	Modified Block 1995 Revision of the Health Habits and History Questionnaire	Age, years of education, total energy, sex, ApoE 𝜀4, stroke, coronary heart disease, and depressive symptoms

Vercambre et al. (2009)	France	Cohort	4,809	13.0	Recent cognitive impairment, 598	NR/76-82	Observed Cognitive Deterioration Scale	Fruit and vegetable	Diet history questionnaire	Age, education level, BMI, physical activity, daily energy intake, smoking, supplement of vitamin D and/or Ca, supplement of other vitamins or minerals, use of postmenopausal hormones, history of depression, history of cancer, history of CHD, history of stroke, history of diabetes mellitus, history of hypertension, and history of hypercholesterolaemia

Wu et al. (2011)	Taiwan, China	Cross-sectional	2,119	–	Cognitive impairment, 472	73.3/≥65	MMSE	F+V	Questionnaire for lifestyle	Age, gender, educational level, marital status, social support, hyperlipidemia, stroke, physical function, depressive symptoms, self-rated health, cigarette smoking, leisure-time physical activity, coffee intake, tea intake, multivitamin intake, and BMI.

*NR, not reported; DSM-IV, Diagnostic and Statistical Manual of Mental Disorders, Fourth Edition; NINCDS-ADRDA, National Institute of Neurological and Communicative Diseases and Stroke-Alzheimer Disease and Related Disorders Association; F+V, fruit and vegetable; FFQ, food frequency questionnaire; BMI, body mass index; CSI-D, Community Screening Instrument for Dementia; PASE, Physical activity Scale for the Elderly; ADL, Activities of Daily Living; GDS, Geriatric Depression Scale; DM, Diabetes; CVD, Cardiovascular Diseases; MMSE, Mini-Mental State Examination; CDR, Clinical Dementia Rating Scale; CHD, coronary heart diseases.*

### Meta-Analysis

**FIGURE 2:**
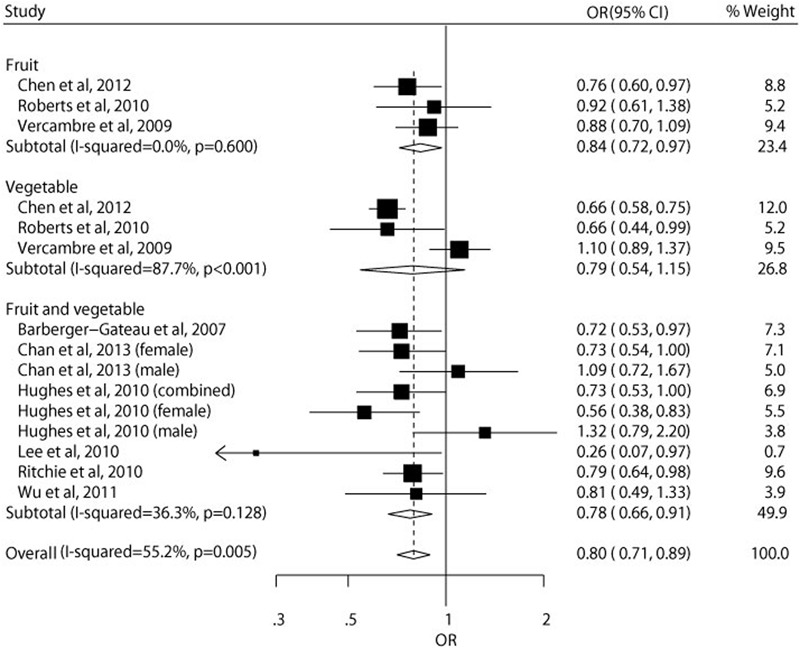
**Relative risk of cognitive impairment and dementia according to the highest vs. lowest category of fruit and vegetables consumption**.

**FIGURE 3:**
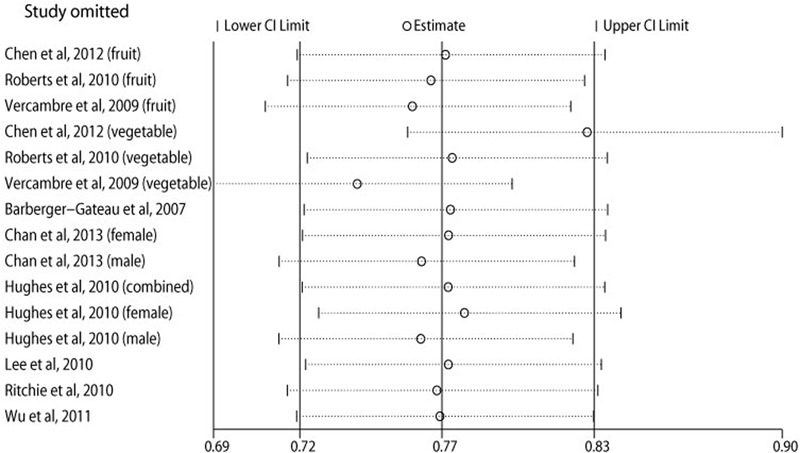
**Sensitivity analysis.** Relative risk of cognitive impairment and dementia according to the highest vs. lowest category of fruit and vegetables consumption by omitting one study in turn.

**Figure 2** shows the results of meta-analysis of relative risk according to the highest vs. lowest category of fruit and vegetable consumption. The summary result showed that high fruit and vegetable consumption was associated with a reduced risk of cognitive impairment and dementia (OR = 0.80, 95% CI 0.71–0.89). A significant heterogeneity was observed for the pooled analyses (*I*^2^ = 55.2%, *P* = 0.005). Subgroup analyses showed that the inverse association between fruit and vegetable consumption and risk of cognitive impairment and dementia was only found in the participants with mean age over 65 years (OR = 0.80, 95% CI 0.71–0.91) and combined sexes (OR = 0.71, 95% CI 0.66–0.78), and studies from the United States and with full marks of study quality score did not show statistical significances (**Table 2**). Sensitivity analysis indicated that the inverse association was not materially changed in the leave-one-out analyses by omitting one study in turn, with a pooled OR of cognitive impairment and dementia range from 0.74 (95% CI 0.69–0.80) to 0.83 (95% CI 0.76–0.90) for the highest vs. lowest category of fruit and vegetable consumption (**Figure 3**).

**Table 2:** Subgroup analysis for studies included in the analysis.

Subgroup analysis	Pooled OR (95% CI), *P*-value for the heterogeneity *Q* test, *I*^2^ statistics (%), number of estimates in included studies (*n*)	
	*n*	Risk estimates of cognitive impairment and dementia
**Mean age**
≥65 years	12	0.80 (0.71–0.91); *I*^2^ = 54.7, *P* = 0.012
< years	3	0.79 (0.51–1.21); *I*^2^ = 70.8, *P* = 0.032
**Sex**
Female	4	0.82 (0.64–1.06); *I*^2^ = 71.9, *P* = 0.014
Male	2	1.18 (0.85–1.63); *I*^2^ = 0.0, *P* = 0.571
Combined	9	0.71 (0.66–0.78); *I*^2^ = 0.0, *P* = 0.567
**Geographic location**
Europe	7	0.84 (0.70–0.99); *I*^2^ = 60.8, *P* = 0.018
United States	2	0.78 (0.58–1.04); *I*^2^ = 21.9, *P* = 0.258
China	6	0.71 (0.64–0.78); *I*^2^ = 38.0, *P* = 0.153
**Study design**
Cohort	9	0.80 (0.69–0.92); *I*^2^ = 67.5, *P* = 0.002
Cross-sectional	6	0.80 (0.67–0.95); *I*^2^ = 23.5, *P* = 0.257
**Study quality score^a^**
Full marks	7	0.91 (0.81–1.02); *I*^2^ = 28.1, *P* = 0.214
Not full marks	8	0.71 (0.65–0.77); *I*^2^ = 40.9, *P* = 0.106
**Disease type**
Cognitive impairment	10	0.82 (0.72–0.93); *I*^2^ = 57.8, *P* = 0.011
Dementia	5	0.73 (0.54–0.83); *I*^2^ = 56.9, *P* = 0.054
**Dietary assessment method**
FFQ	3	0.79 (0.65–0.96); *I*^2^ = 30.2, *P* = 0.239
Others	12	0.79 (0.69–0.91); *I*^2^ = 61.2, *P* = 0.003

*OR, odds ratio; CI, confidence interval; FFQ, food frequency questionnaire.*

*^a^Full mark was defined as nine points for cohort studies and five points for cross-sectional studies based on the Newcastle–Ottawa Scale.*

### Dose–Response Meta-Analysis

**FIGURE 4:**
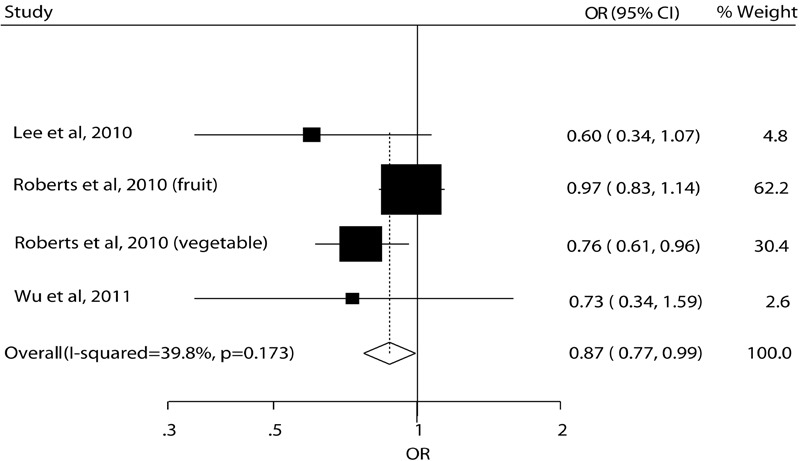
**Dose-response meta-analysis.** Relative risk of cognitive impairment and dementia for an increment of 100 g per day of fruit and vegetables consumption.

Only four estimates from three individual studies were included in the dose–response meta-analysis, because there were only two categories of fruit and vegetable consumption in other studies, and dose–response meta-analysis requires data for the distribution of cases and person-time across at least three categories of exposure (Alexander et al., 2009). In the dose–response meta-analysis, the reduced risk of cognitive impairment and dementia by an increment of 100 g per day of fruit and vegetable consumption was only observed in the estimate for vegetable in Roberts et al.’s study. However, the pooled result showed that an increment of 100 g per day of fruit and vegetable consumption was associated with a reduced risk of cognitive impairment and dementia (OR = 0.87, 95% CI 0.77–0.99) (**Figure 4**). There was no potential heterogeneity among studies (*I*^2^ = 39.8%, *P* = 0.173).

### Publication Bias

**FIGURE 5:**
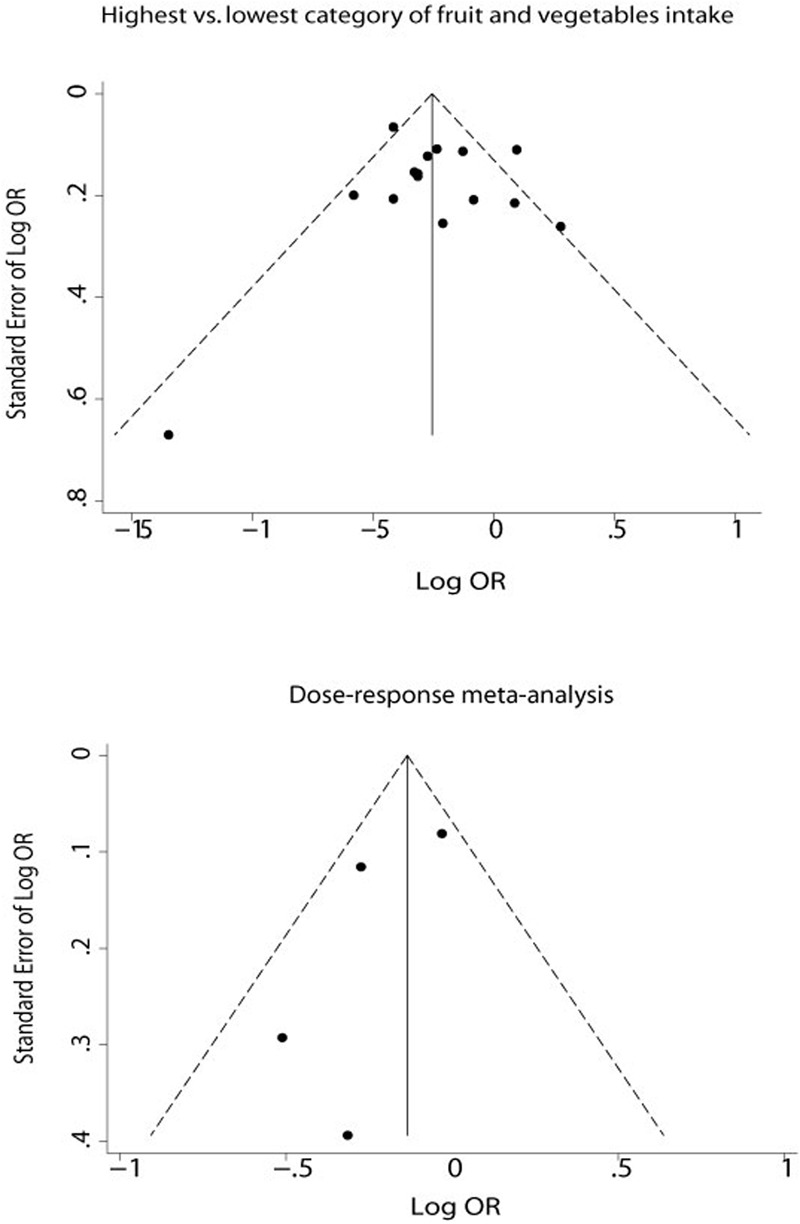
**Funnel plot to explore publication bias.** The vertical line is at the mean effect size.

Visual assessment of funnel plots (**Figure 5**) showed that the studies were distributed fairly symmetrically about the combined effect size in both the meta-analysis and the dose–response meta-analysis, which suggests little publication bias. Egger’s regression test (*P* = 0.624 and *P* = 0.273, respectively) and Begg–Mazumdar test (*P* = 0.882 and *P* = 1.000, respectively) also showed that there was no potential publication bias in the meta-analysis and the dose–response meta-analysis.

## Discussion

We have quantitatively assessed the relation between fruit and vegetable consumption and risk of cognitive impairment and dementia through a meta-analysis of existing epidemiological studies. Evidence from the literature is mixed. Some studies indicate that fruit and vegetable intake has a protective effect on global cognitive performance (Nurk et al., 2010) and memory and executive function (Sabia et al., 2009), and people with high intakes of fruit and vegetables score higher on cognitive tests and are less likely to have cognitive decline and lower incidence of dementia (Gillette Guyonnet et al., 2007), whereas others suggest nonsignificant effects of fruit and vegetable intake on cognitive flexibility, memory, and information processing speed (Nooyens et al., 2011); perceptual speed and attention (Morris et al., 2006); or verbal memory, general cognition, working memory, and category fluency (Kang et al., 2005). Overall, measurement limitations, as well as the type of cognitive function measured, the types of fruit and vegetables consumed, or amounts of fruit and vegetable intake might have led to the inconsistence in the observed results. The value of the current meta-analysis compensates for the individual lack of precision in most of the studies, a problem that was alleviated by pooling the data of all the studies. Therefore, meta-analysis of these studies is a potentially powerful approach to assess the long-term effects of fruit and vegetable consumption on cognitive impairment and dementia risk. Our study shows that an increased consumption of fruit and vegetables is related to a reduced risk of cognitive impairment and dementia. Moreover, we found an increment of 100 g per day of fruit and vegetable consumption was related to an approximately 13% reduction in cognitive impairment and dementia risk.

Our findings extend the results of a previous systematic review (Loef and Walach, 2012), which found increased intake of vegetables was associated with a lower risk of dementia and slower rates of cognitive decline in older age but evidence that this association is also valid for high fruit consumption is lacking. Morris et al. (2006) also found that high vegetable but not fruit consumption may be associated with slower rate of cognitive decline with older age. Interestingly, in our subtotal estimates, we found that fruit and fruit and vegetable combined but not vegetable consumption had a protective effect on cognitive impairment and dementia (**Figure 2**). But in our meta-analysis, most included studies used fruit and vegetable combined as the exposure variable, and there were only three studies included in the subtotal estimates of both fruit alone and vegetable alone, leading to a low statistical power. This is also the reason why we pooled the results of fruit alone, vegetable alone, and fruit and vegetable combined together. More studies are needed to further assess the role of fruit and vegetable separately on the risk of cognitive impairment and dementia. In addition, our meta-analysis restricted to overall fruit and vegetable consumption, some particular kinds of fruit and vegetable intake, such as nuts, cruciferous vegetables, dark and green leafy vegetables, root vegetables, cabbage, tomatoes, soybeans, and soybean products (Kang et al., 2005; Gu et al., 2010; Nooyens et al., 2011; Ozawa et al., 2013), may also have a protective role on cognitive impairment and dementia risk, but they were not included in our meta-analysis due to the limited data. More investigations focusing on the association between these specific fruits and vegetables and cognitive impairment and dementia risk are also urgently needed.

Epidemiological and animal investigations support the protective effect of fruit and vegetables against cognitive impairment and dementia. The brain is extremely susceptible to oxidative damage (Floyd and Hensley, 2002), and fruits and vegetables are high in antioxidants (Proteggente et al., 2002). Some epidemiological studies (Engelhart et al., 2002; Morris et al., 2002; Grodstein et al., 2003; Zandi et al., 2004) have reported that high intake of antioxidants is related to reduced cognitive decline or dementia. Furthermore, animal studies have indicated that antioxidants prevent neuronal damage (Maneesub et al., 1993; Yokota et al., 2001) and improve cognitive performance (Sack et al., 1996; Cotman et al., 2002). A study from the rats model suggests that the antioxidant vitamins found in green leafy vegetables could be particular important for cognitive health (Joseph et al., 1999). Epidemiological studies (Ebly et al., 1998; Lindeman et al., 2000; Seshadri et al., 2002) has also found that folate in some fruits and vegetables is linked to cognitive function and dementia. Folate deficiency can increase the level of homocysteine, which has direct neurotoxic effects in cell lines and animal models (Lipton et al., 1997; Kruman et al., 2002). In contrast, other facts are not in favor of a causal relation. Generally, fruit and vegetable consumers have healthier lifestyles and dietary pattern, which themselves are associated with better cognitive performance (Evans et al., 1997; Kesse-Guyot et al., 2012). Although most studies have adjusted for lifestyle factors, residual confounders may still explain part of the favorable association with cognitive performance and dementia. High consumptions of fruit and vegetables are associated with a prudent diet pattern, and inversely related to the intake of saturated fat-rich food (Tucker et al., 2005), which may also contribute to the lower risk of cognitive impairment and dementia (Kalmijn et al., 1997). Therefore, the results of current study support the concept that the regular intake of fruit and vegetables is associated with reduced risk of cognitive impairment and dementia, however, it does not establish a causal relation. Well designed randomized controlled trials that address a specific mechanism of fruit and vegetables consumption and reduced risk of cognitive impairment and dementia are needed.

In subgroup analyses, we found that there was a statistically significant inverse association between fruit and vegetable consumption and cognitive impairment and dementia risk in participants with mean age over 65 years, but not in those with mean age less than 65 years. This may be because that the incidence of cognitive impairment and dementia increases sharply with the age grows. Thus, the protective effects of fruit and vegetables against cognitive impairment and dementia are more prominent among the older populations. Notably, the null inverse association in younger participants might also result from the limited number of included studies. More studies are warranted to investigate the potential difference between the different age groups. In the subgroup analysis of geographic location, we found the pooled OR for China was much smaller than that for Europe and the United States (0.71 with 95% CI 0.64–0.78, 0.84 with 95% CI 0.64–0.78, and 0.78 with 95% CI 0.58–1.04 for China, Europe, and the United States, respectively). This means that the protective effects of fruit and vegetables against cognitive impairment and dementia are stronger for Chinese populations than the Western populations. This may be explained by the different dietary patterns and food preparation methods between Chinese and Western populations. Firstly, the proportion of saturated fat-rich food is higher in the Western dietary pattern, which may increase the risk of cognitive impairment and dementia as discussed above. Secondly, in Western world, people mainly eat raw vegetables, such as in the form of salad, while the Chinese populations often eat cooked, steamed, or boiled vegetables. Usually, people tend to eat more vegetables after being cooked, and the cooked vegetables can be more easily digested because the cell wall of raw vegetables is relatively harder, which will increase the burden of digestion. In addition, studies show that the nutritional quality increases in all cooked vegetables because of matrix softening and increased extractability of compounds, which could be partially converted into more antioxidant chemical species (Miglio et al., 2008; Porrini and Riso, 2008).

The strengths of the present meta-analysis include the considerable number of studies and subjects included, as well as the acceptable methodologic quality of the studies on which the analysis is based. The study has several limitations. Firstly, a substantial heterogeneity across studies was apparent in the meta-analysis. The heterogeneity was not accounted by age, sex, geographic location, study design, disease type, and dietary assessment method. However, the subgroup analysis for study quality score reduced the heterogeneity, which means the study quality is a main source of heterogeneity. Secondly, the assessment for fruit and vegetable consumption is mostly based on self-reported habits, and such data are subject to recall errors. In addition, there were many other differences among studies, including dietary assessment methods, the variety of fruit or vegetables investigated, the definition of the reference group, and the choice of exposure categories. These differences could affect the estimation of the true relation. Thirdly, the meta-analysis is based on observational studies, which leaves the possibility that potential confounders cannot be ruled out, affect the relation between fruit and vegetable consumption and risk of cognitive impairment and dementia. A meta-analysis is not able to solve problems with confounding that may be inherent in the included studies. However, most studies have made adjustment of major confounding factors, which should reduce the potential bias due to the dietary and lifestyle factors. Finally, due to the limited studies, we combined consumption of fruit and vegetable together as the exposure variable and cognitive impairment and dementia together as the outcome. The effects of fruit and vegetable separately on cognitive impairment or dementia should be further investigated in future studies.

## Conclusion

This meta-analysis indicates significant inverse association between fruit and vegetable consumption and risk of cognitive impairment and dementia. The risk of cognitive impairment and dementia was reduced by 20% for a higher consumption of fruit and vegetables, and by 13% for an increment of 100 g per day of fruit and vegetable consumption. For further studies, based on our findings, we suggest that the investigators should improve the standardization of various dietary assessment methods, which may make the results more accurate and conceivable. Furthermore, the use of genetic and biological makers as surrogate end points in the future studies should help to clarify the cause and effect relationship that link fruit and vegetable consumption and cognitive impairment and dementia.

## Author Contributions

ZZ designed the study. JH, DS, and RD collected the data. XJ performed all analyses. XJ, JH, JW, and ZZ wrote the manuscript. All authors contributed to writing of this manuscript.

## Conflict of Interest Statement

The authors declare that the research was conducted in the absence of any commercial or financial relationships that could be construed as a potential conflict of interest.
