# Supplementary material for: Increased Consumption of Fruit and Vegetables Is Related to a Reduced Risk of Cognitive Impairment and Dementia: Meta-Analysis
Source: Front Aging Neurosci. 2017 Feb 7;9:18. doi: 10.3389/fnagi.2017.00018 (PMC5293796; doi:10.3389/fnagi.2017.00018)
Supplement: Supplementary file 1 [file Table_1.DOCX]

**Table S1: MOOSE Checklist**

| **Criteria** | | **Brief description of how the criteria were handled in the meta-analysis** |
| --- | --- | --- |
| **Reporting of background should include** | |  |
| √ | Problem definition | Previous studies find a favorable relation between fruit and vegetable consumption and risk of dementia or cognitive decline, although sometimes the results are inconsistent. Moreover, the strength of the favorable relation remains uncertain due to the differences in sample selections, methodological approaches, analytical techniques, and outcome definitions. |
| √ | Hypothesis statement | Fruit and vegetables are high in antioxidants, vitamins and folate, and these micronutrients have been related to cognitive benefits in epidemiological and laboratory studies. |
| √ | Description of study outcomes | Cognitive impairment and dementia |
| √ | Type of exposure or intervention used | Fruit and vegetables consumption |
| √ | Type of study designs used | Observational study |
| √ | Study population | Elderly with cognitive impairment or dementia and controls |
| **Reporting of search strategy should include** | |  |
| √ | Qualifications of searchers (eg, librarians and investigators) | The credentials of the two investigators JH and DS are provided in the author list. |
| √ | Search strategy, including time period included in the synthesis and keywords | Medline, Embase, and Cochrane Library electronic databases (from 1970 to January 2016).  Keywords: “Fruit”, “Vegetables”, “Mild Cognitive Impairment”, “Dementia”, “cognitive decline”, “cognitive impairment”, “survey”, and “Data Collection”. |
| √ | Effort to include all available studies, including contact with authors | The references of all retrieved articles and recent reviews were also manually reviewed. |
| √ | Databases and registries searched | Medline, Embase, and Cochrane Library electronic databases |
| √ | Search software used, name and version, including special features used (eg, explosion) | We did not employ a special search software. |
| √ | Use of hand searching (eg, reference lists of obtained articles) | References of all retrieved articles and recent reviews were reviewed. |
| √ | List of citations located and those excluded, including justification | Details of the literature search process are outlined in the flow chart. |
| √ | Method of addressing articles published in languages other than English | We placed restrictions on English. |
| √ | Method of handling abstracts and unpublished studies | No attempt was made to find articles in languages other than English or to contact authors of unpublished works. |
|  | Description of any contact with authors. | - |
| **Reporting of methods should include** | |  |
| √ | Description of relevance or appropriateness of studies assembled for assessing the hypothesis to be tested | The inclusion criteria are presented in the “Search strategy and eligibility criteria” section. |
| √ | Rationale for the selection and coding of data (eg, sound clinical principles or convenience) | Two researchers (DS and RD) independently extracted the following data from each publication: author, country, study design, sample size, disease type (cognitive impairment or dementia), number of cases, age, disease ascertainment, exposure variable (fruit, vegetable, or fruit and vegetable), exposure assessment, risk estimates with CIs, and factors adjusted for. The most adjusted estimate was included when a study reported more than one risk estimate. |
| √ | Documentation of how data were classified and coded (eg, multiple raters, blinding, and inrerrater reliability) | Data were independently extracted and analyzed by two investigators (JH and XJ) and final decision was reached by consensus. |
| √ | Assessment of confounding (eg, comparability of cases and controls in studies where appropriate) | Table 1 presents the adjustment factors for each study. |
| √ | Assessment of study quality, including blinding of quality assessors; stratification or regression on possible predictiors of study results | The quality of each study was assessed by two investigators (DS and RD), using the Newcastle-Ottawa Scale. |
| √ | Assessment of heterogeneity | The *Q*-statistic and *I*-squared (*I*^2^) statistic were used to explore the heterogeneity among studies. |
| √ | Description of statistical methods (eg, complete description of fixed or random effects models, justification of whether the chosen models account for predictors of study results, dose-response models, or cumulative meta-analysis) in sufficient detail to be replicated | Description of methods of meta-analyses, subgroup analyses, dose-response meta-analysis, sensitivity analysis, and assessment of publication bias are detailed in the “Statistical analysis” section. |
| √ | Provision of appropriate tables and graphics | Two main tables and four supplemental tables are provided. One flow chart and three forest plots appear in the main text. |
| **Reporting of results should include** | |  |
| √ | Graph summarizing individual study estimates and overall estimate | Figures 2, 4 |
| √ | Table giving descriptive information for each study included | Table 1 |
| √ | Results of sensitivity testing (eg, subgroup analysis) | “Results” section; Figure 3, Table 2 |
| √ | Indication of statistical uncertainty of findings | 95% confidence intervals were presented with all summary effect estimates. |
| **Reporting of discussion should include** | |  |
| √ | Quantitative assessment of bias (eg, publication bias) | “Results” section, and Figure 5. |
| √ | Justification for exclusion (eg, exclusion of non-English-language citations) | The details of the exclusion of studies are shown in Flow chart. |
| √ | Assessment of quality of included studies | Studies have been subgroup analyzed by the quality. |
| **Reporting of conclusions should include** | |  |
| √ | Consideration of alternative explanations for observed results | We discussed that the meta-analysis is based on observational studies, thus, we cannot exclude chance, residual or unmeasured confounding as alternative explanation for our findings. |
| √ | Generalization of the conclusions (ie, appropriate for the data presented and within the domain of the literature review) | We discussed that the results of current study support the concept that the regular intake of fruit and vegetables is associated with reduced risk of cognitive impairment and dementia, however, it does not establish a causal relation |
| √ | Guidelines for future research | We discussed that for further studies, based on our findings, we suggest that the investigators should improve the standardization of various dietary assessment methods, which may make the results more accurate and conceivable. |
| √ | Disclosure of funding source | The authors received no specific funding for this work. |
