# Supplementary material for: Increased Consumption of Fruit and Vegetables Is Related to a Reduced Risk of Cognitive Impairment and Dementia: Meta-Analysis
Source: Front Aging Neurosci. 2017 Feb 7;9:18. doi: 10.3389/fnagi.2017.00018 (PMC5293796; doi:10.3389/fnagi.2017.00018)
Supplement: Supplementary file 2 [file Table_2.DOCX]

**Table S2:** Categories of fruit and vegetable consumption and the conversions of categories

| Reference | Exposure variable | Original categories | Conversional categories^a^ |
| --- | --- | --- | --- |
| Lee et al, 2010 | F+V | <1 serving per day | 39.3 g/day |
|  |  | 1 to 2 servings per day | 117.8 g/day |
|  |  | 2 or more servings per day | 196.3 g/day |
| Roberts et al, 2010 | Fruit | ≤153.4 g/day | 76.7 g/day |
|  |  | 153.5-276.8 g/day | 215.2 g/day |
|  |  | >276.8 g/day | 338.5 g/day |
|  | Vegetable | ≤109.6 g/day | 54.8 g/day |
|  |  | 109.7-191.0 g/day | 150.4 g/day |
|  |  | >191.0 g/day | 231.7 g/day |
| Wu et al, 2011 | F+V | Low frequencies (two times or less in fruits and five times or less in vegetables in a week ) | 19.5 g/day |
|  |  | Medium frequencies | 58.7 g/day |
|  |  | High frequencies (a daily basis for both fruits and vegetables) | 78.5 g/day |

*Abbreviations:* F+V, fruit and vegetables

^a^ If the original study expressed fruit and vegetable consumption in serving, we assume that one serving of fruit is 80 g and one serving of vegetable is 77 g. We converted the level of consumption category based on the calculated midpoint of fruit and vegetable consumption if the study did not report the median of exposure category.
