# Supplementary material for: Increased Consumption of Fruit and Vegetables Is Related to a Reduced Risk of Cognitive Impairment and Dementia: Meta-Analysis
Source: Front Aging Neurosci. 2017 Feb 7;9:18. doi: 10.3389/fnagi.2017.00018 (PMC5293796; doi:10.3389/fnagi.2017.00018)
Supplement: Supplementary file 3 [file Table_3.DOCX]

**Table S3: Quality assessment of the included studies (cohort studies)**

| Study | Selection | | | | Comparability | Outcome | | | Overall quality assessment score (of a maximum of 9) |
| --- | --- | --- | --- | --- | --- | --- | --- | --- | --- |
|  | Representativeness of the exposed cohort | Selection of the non exposed cohort | Ascertainment of exposure | Demonstration that outcome of interest was not present at start of study | Comparability of cohorts on the basis of the design or analysis | Assessment of outcome | Was follow-up long enough for outcomes to occur | Adequacy of follow up of cohorts |  |
| Barberger-Gateau et al, 2007 | * Truly representative of the average population in the community | * Drawn from the same community as the exposed cohort | * Structured interview | * The study demonstrated that dementia was not present at start of study | ** Study controls for age, gender, education, city, income, marital status, ApoE genotype, body mass index and diabetes | * Independent blind assessment | * The study select an adequate follow up period for outcome of interest | Subjects lost to follow up might introduce bias (79.6% follow up) | 8 |
| Chen et al, 2012 | * Truly representative of the average population in the community | * Drawn from the same community as the exposed cohort | * Structured interview | * The study demonstrated that cognitive decline was not present at start of study | * Study controls for age, gender, marital status, financial status, residential area, BMI, hypertension, diabetes, smoking, alcohol, tea drinking, and exercise habits in some analyses | * Independent blind assessment | The study does not select an adequate follow up period for outcome of interest | * Subjects lost to follow up might not introduce bias (82.3% follow up) | 7 |
| Hughes et al, 2010 | * Truly representative of the average population in the community | * Drawn from the same community as the exposed cohort | * Structured interview | * The study demonstrated that dementia or AD was not present at start of study | ** Study controls for age at cognitive screening, gender, education, smoking, alcohol drinking, angina pectoris, BMI, total food compared to others, marital status, and exercise | * Independent blind assessment | * The study select an adequate follow up period for outcome of interest | Subjects lost to follow up might introduce bias (66.4% follow up) | 8 |
| Ritchie et al, 2010 | * Somewhat representative of the average population in the community | * Drawn from the same community as the exposed cohort | * Structured interview | * The study demonstrated that mild cognitive impairment or dementia was not present at start of study | * Study controls for age and sex | * Independent blind assessment | * The study select an adequate follow up period for outcome of interest | * Subjects lost to follow up might not introduce bias (100% follow up) | 8 |
| Vercambre et al, 2009 | * Truly representative of the average population in the community | * Drawn from the same community as the exposed cohort | * Structured interview | * The study demonstrated that recent cognitive decline or dementia was not present at start of study | ** Study controls for age, education level, BMI, physical activity, daily energy intake, smoking, supplement of vitamin d AND/OR Ca, supplement of other vitamins or minerals, use of postmenopausal hormones, history of depression, history of cancer, history of CHD, history of stroke, history of diabetes mellitus, history of hypertension, and history of hypercholesterolaemia | * Independent blind assessment | * The study select an adequate follow up period for outcome of interest | * Subjects lost to follow up might not introduce bias (100% follow up) | 9 |
