# Supplementary material for: Increased Consumption of Fruit and Vegetables Is Related to a Reduced Risk of Cognitive Impairment and Dementia: Meta-Analysis
Source: Front Aging Neurosci. 2017 Feb 7;9:18. doi: 10.3389/fnagi.2017.00018 (PMC5293796; doi:10.3389/fnagi.2017.00018)
Supplement: Supplementary file 4 [file Table_4.DOCX]

**Table S4: Quality assessment of the included studies (cross-sectional studies)**

| Study | Selection | | Comparability | Exposure | Overall quality assessment score (of a maximum of 5) |
| --- | --- | --- | --- | --- | --- |
|  | Representativeness of the sample | Ascertainment of exposure | Comparability of groups on the basis of the design or analysis | Assessment of outcome |  |
| Chan et al, 2013 | * Truly representative of the average population in the community | * Structured interview | * * Study controls for age, BMI, PASE, energy intake, educational level, Hong Kong ladder, community ladder, smoking status, alcohol use, on. Of ADLs, GDS category, self-reported history of DM, hypertension, and CVD/stroke | * Independent blind assessment | 5 |
| Lee et al, 2010 | * Somewhat representative of the average population in the community | * Structured interview | * Study controls for age, sex, and education | * Independent blind assessment | 4 |
| Roberts et al, 2010 | * Truly representative of the average population in the community | * Structured interview | * * Study controls for age, years of education, total energy, sex, ApoE ε4, stroke, coronary heart disease, and depressive symptoms | * Independent blind assessment | 5 |
| Wu et al, 2011 | * Truly representative of the average population in the community | * Structured interview | * * Study controls for age, gender, educational level, marital status, social support, hyperlipidemia, stroke, physical function, depressive symptoms, self-rated health, cigarette smoking, leisure-time physical activity, coffee intake, tea intake, multivitamin intake, and BMI. | * Independent blind assessment | 5 |
